# A Hybrid Model for Coronavirus Disease 2019 Forecasting Based on Ensemble Empirical Mode Decomposition and Deep Learning

**DOI:** 10.3390/ijerph20010617

**Published:** 2022-12-29

**Authors:** Shidi Liu, Yiran Wan, Wen Yang, Andi Tan, Jinfeng Jian, Xun Lei

**Affiliations:** 1 School of Public Health and Management, Chongqing Medical University, Chongqing 400016, China; 2 Research Center for Medicine and Social Development, Chongqing Medical University, Chongqing 400016, China; 3 Collaborative Innovation Center of Social Risks Governance in Health, Chongqing Medical University, Chongqing 400016, China; 4 Research Center for Public Health Security, Chongqing Medical University, Chongqing 400016, China; 5 International Business School, Yunnan University of Finance and Economics, No. 237, Longquan Road, Kunming 650221, China

**Keywords:** COVID-19 prediction, deep learning, long short-term memory, ensemble empirical mode decomposition

## Abstract

Background: The novel coronavirus pneumonia that began to spread in 2019 is still raging and has placed a burden on medical systems and governments in various countries. For policymaking and medical resource decisions, a good prediction model is necessary to monitor and evaluate the trends of the epidemic. We used a long short-term memory (LSTM) model and the improved hybrid model based on ensemble empirical mode decomposition (EEMD) to predict COVID-19 trends; Methods: The data were collected from the Harvard Dataverse. Epidemic data from 21 January 2020 to 25 April 2021 for California, the most severely affected state in the United States, were used to develop an LSTM model and an EEMD-LSTM hybrid model, which is an LSTM model combined with ensemble empirical mode decomposition. In this study, ninety percent of the data were adopted to fit the models as a training set, while the subsequent 10% were used to test the prediction effect of the models. The mean absolute percentage error, mean absolute error, and root mean square error were used to evaluate the prediction performances of the models; Results: The results indicated that the number of confirmed cases in California was increasing as of 25 April 2021, with no obvious evidence of a sharp decline. On 25 April 2021, the LSTM model predicted 3666418 confirmed cases, whereas the EEMD-LSTM predicted 3681150. The mean absolute percentage errors for the LSTM and EEMD-LSTM models were 0.0151 and 0.0051, respectively. The mean absolute and root mean square errors were 5.58 × 10^4^ and 5.63 × 10^4^ for the LSTM model and 1.9 × 10^4^ and 2.43 × 10^4^ for the EEMD-LSTM model, respectively; Conclusions: The results showed the advantage of an EEMD-LSTM model over a single LSTM model, and the established EEMD-LSTM model may be suitable for monitoring and evaluating the epidemic situation and providing quantitative analysis evidence for epidemic prevention and control.

## 1. Introduction

Coronavirus Disease 2019 (COVID-19) is an acute respiratory viral disease characterized by fever, tiredness, and a dry cough. Acute respiratory distress syndrome, septic shock, multiple organ failure, and even death can occur in severe cases [1]. The first case of COVID-19 was reported from Wuhan, China, in December 2019. Following that, cases were recorded and reported globally. There has been a surge in coronavirus cases globally. Severe acute respiratory syndrome coronavirus 2, in particular, has caused a serious outbreak in the United States. As of 8 April 2022, the number of confirmed cases had surpassed 8 million, and more than 900,000 deaths have been reported [2]. The variants of the novel coronavirus have also contributed to a surge in infections globally, although vaccines have emerged. As a consequence, pandemic containment remains a public health challenge, and the lives of people are in danger, especially those who lack access to medical resources. There is no doubt that epidemic prediction models can improve our understanding of the pandemic and provide evidence for measures to contain it.

Since the outbreak of the epidemic, the traditional dynamics models, including the susceptible-infected-recovered-deaths model and its modifications, have been widely applied to the simulation of the epidemic and related predictions [3,4,5]. It was challenging to use the models because of the difficulty in estimating parameters, underlying nonlinear system of ordinary differential equations, lack of access to some real data, and assumptions related to them [6]. ArunKumar explored the use of autoregressive integrated moving average (ARIMA) models for COVID-19 prediction, which have been used for epidemic predictions for decades and proven to have several advantages related to prediction [7]. However, the ARIMA model was essentially a linear model that was not good at capturing nonlinear trends. To overcome the barriers of these approaches, deep learning algorithms were used to identify the non-linear disease patterns. Regarding nonlinear data, deep learning models have better learning and predictive abilities. Moreover, the COVID-19 is a time series data set and it is highly recommended to use sequential networks to extract the patterns from it. Among deep learning models, the long short-term memory (LSTM) algorithm is appropriate for sequential learning tasks [8]. Based on its excellent capacity, the LSTM algorithm can learn numerous information from data. To make use of sequential data for prediction using deep learning, Yudistira used an LSTM model to learn the correlation on COVID-19 data over time [9]. Some researchers have attempted to develop LSTM models to accurately predict the epidemic trends [10,11,12]. LSTM models have demonstrated some advantages over traditional deep learning algorithms; however, they occasionally show poor performance in predictions, compared with other algorithms [13]. To improve accuracy, several researchers have modified the LSTM algorithm. The W-LSTM model performed better than previously published models, including ARIMA and LSTM, based on model performance evaluation criteria such as the mean square error and mean absolute percentage error (MAPE) [14]. The proposed forecast models, including ARIMA, support vector regression, LSTM, and bidirectional LSTM, have been used for time series predictions of confirmed cases, deaths, and recoveries in ten major countries affected by COVID-19 [13]. Rasjid et al. aimed to predict the COVID-19 death and infection rates in Indonesia using the Savitzky Golay Smoothing and LSTM model [15]. In the present study, we have developed a model that combines LSTM and ensemble empirical mode decomposition (EEMD) to improve accuracy.

Empirical mode decomposition (EMD) is an adaptive time-space analysis method suitable for processing series that are non-stationary and non-linear [16]. It decomposes an original dataset into a series of intrinsic mode functions (IMFs) and a residual function, revealing unique features in the data. Mode mixing, on the other hand, is one of the major challenges with empirical mode decomposition, and it is often known as a single IMF with components of several scales or a distinct IMF composed of identical components of other scales. Wu and Huang presented an improved EMD approach, which is EEMD, to overcome the mode mixing in the typical EMD [17]. EEMD is a noise-assisted data analysis method that effectively eliminates the problem of mode mixing by introducing white noise into the original data. According to a report [18], the decomposition strategy also enhances prediction accuracy by supplying explicit signals as model input. A hybrid EEMD-LSTM approach has been used for useful predictions in the fields of precipitation and agricultural product price [19,20]. However, little is known about the value of the EEMD-LSTM in predicting the COVID-19 epidemic trends.

Severe acute respiratory syndrome coronavirus 2 caused a significant outbreak in the United States. California had the most confirmed cases in the United States as of 25 April 2021 [21]. California is a major state in the western (Pacific) area of the United States and has the second-largest metropolis (Los Angeles). It is also the most populous state in the United States and a major global economic hub [22]. As a result, we take the lead in model fitting using California data.

Based on previous studies, the goals of this research were to propose a hybrid EEMD-LSTM model for COVID-19 epidemic prediction and compare a single LSTM model with the proposed hybrid EEMD-LSTM model based on the testing.

## 2. Materials and Methods

### 2.1. Study Design and Data

Study design: Public data mining and predictive modeling. This was a forecasting model study to compare the prediction performance of different models and predict the pandemic trends more accurately.

The U.S. state epidemic data (cumulative confirmed cases, deaths) from 21 January 2020, to 25 April 2021, were obtained from the Harvard Dataverse [21].

The cumulatively confirmed cases in America were visualized using R version 4.0.5 (31 March 2021). Ninety percent of the data were used for the training set and the remaining 10% were used as the test set. To make the neural network model converge faster, the min-max normalization was used to normalize the data to the interval of 0 and 1. Assuming that $x_{i}$ comes from the sequence, x, normalization was performed as follows:

(1) $$x_{i}{}^{*}=\frac{x_{i}-x_{\min}}{x_{\max}-x_{\min}},i=1,2,\cdots n$$

### 2.2. Study Area

Covering an area of 423,970 sq. km, California is a large state in the western (Pacific) region of the United States that is home to one of the most diverse populations globally. It contains the second largest city (Los Angeles), 3 of the largest 10 cities (Los Angeles, San Diego, and San Jose), and the largest county (Los Angeles County) in the United States. In addition, its population was approximately 39,995,077 in 2022. California is the 3rd largest state by area, which places its population density at 251.3 per square mile [22].

### 2.3. Empirical Mode Decomposition, EMD

Empirical mode decomposition, which was proposed by Huang et al. [16], is suited to nonlinear and non-stationary data through signal decomposition. It is used to decompose complex raw time-series data into a series of IMFs and a residue. The intrinsic mode function captures the repeating behavior of the signal for a given particular time scale and keeps the full non-stationary information of the raw data under consideration. Any non-stationary and non-linear time series is made up of different simple intrinsic modes of oscillation, according to the empirical mode decomposition approach. The essence of the method is to objectively detect these intrinsic oscillatory modes in the data based on their characteristic time scales and deconstruct the data accordingly. Most of the riding waves, such as oscillations with no zero-crossing between extrema, can be avoided using a technique known as sifting. As a result, the empirical mode decomposition method takes signal oscillations into account at a very local level and divides the data into locally non-overlapping time scale components [23]. It decomposes a signal *x*(*t*) into its constituent IMFs, each of which meets the following two conditions: (1) in the entire data set, the number of extrema and the number of zero crossings must either be equal or differ at most by one; (2) at any point, the mean value of the envelope defined by the local maximum and minimum is zero.

### 2.4. Ensemble Empirical Mode Decomposition, EEMD

The EMD is limited by the large length of the time series [23]. More importantly, it has shown mode mixing where the original data appeared at a high frequency [17]. To solve these problems, Huang et al. proposed EEMD. Ensemble empirical mode decomposition first adds Gaussian white noise to the original sequence and uses EMD to decompose the sequence into a series of IMFs. For any sequence, X(t), the EEMD steps are as follows:

- First, the initial parameters, such as the times of the ensembles and the variance of the added white noise, are set, and white noise is added to the sequence, X(t).
- Second, the local extremum of the sequence, X(t), is extracted.
- Third, the upper envelope, $e_{u}(t)$, and the lower envelope, $e_{l}(t)$, of the sequence, X(t), are determined.
- Fourth, the mean values of the upper and lower envelope are calculated using the following formula: $m(t)=\frac{e_{l}(t)+e_{u}(t)}{2}$.
- Fifth, the component is extracted as follows h(t)=X(t)−m(t).
- Sixth, if the component, h(t), satisfies the conditions for the establishment of the IMF, the first IMF ($imf_{1}(t)$) is obtained; otherwise, the above steps are repeated until the conditions are met and the first IMF is obtained.
- Seventh, a new sequence is calculated using $r_{1}=X(t)-imf_{1}(t)$, and the above steps are repeated until all $imf_{j}(t),j=1,2,\cdots n$ and the residual sequence $r_{n}$ are obtained.

### 2.5. Long Short-Term Memory, LSTM

**Figure 1:**
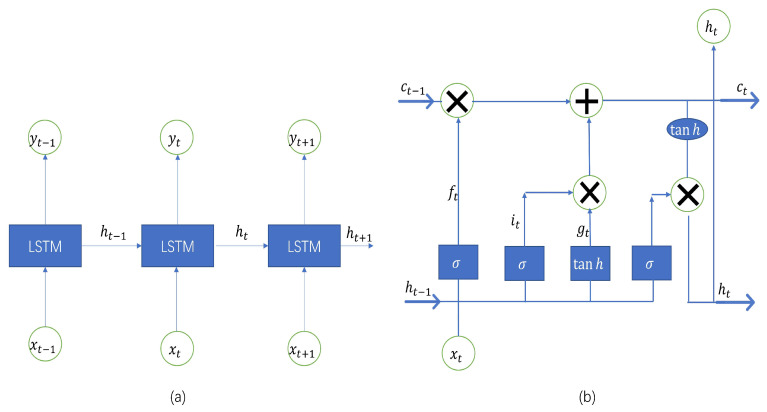
Architecture of long short-term memory [25]. (**a**) Architecture of long short-term memory. (**b**) Architecture of long short-term memory model memory unit.

The LSTM developed by Hochreiter and Schmidhuber is a deep learning time series algorithm based on the recurrent neural network (RNN) framework [24]. It consists of memory modules, and their recurrent neural networks can bridge a large number of discrete time steps through the use of memory cells and gate units. In the hidden layers of the neural network, the LSTM has “cells” that have three gates: input, output, and forget gates. These gates regulate the flow of data required by the network to derive an output. The three gates selectively forget the prior memory, receive part of the new information, and selectively output the information when the memory unit is refreshed. The detailed structure of the LSTM is shown in Figure 1.

The functions of each gate are described as follows. The forget gate removes the data that are no longer relevant in the cell state. The gate receives two inputs, $x_{t}$ (input at the current time) and $h_{t-1}$ (prior cell output), which are multiplied with weight matrices before bias is added. The output of the activation function, which receives the outcome, is binary. The input gate is responsible for adding important information to the cell state. To start, the inputs, $h_{t-1}$ and $x_{t}$, are used to regulate the information using the sigmoid function and filter the values that need to be remembered similar to the forget gate. A vector containing every possible value between $h_{t-1}$ and $x_{t}$ is produced using the tanh function, which produces an output ranging from −1 to +1. To extract useful information, the values of the vector and regulated values are finally multiplied. The role of the output gate is to gather pertinent data from the current cell state and display it as output. The tanh function is first used in the cell to create a vector. The data are subsequently filtered by the values to be remembered using the inputs, $h_{t-1}$ and $x_{t}$, and the derived information is regulated using the sigmoid function. The values of the vector and regulated values are finally multiplied and supplied as input and output to the following cell, respectively [8]. The equations for an LSTM unit are shown as follows,

(2) $$i_{t}=\sigma_{g}\left(W_{i}x_{t}+U_{i}h_{t-1}+b_{i}\right)$$

(3) $$f_{t}=\sigma_{g}\left(W_{f}x_{t}+U_{f}h_{t-1}+b_{f}\right)$$

(4) $$o_{t}=\sigma_{g}\left(W_{o}x_{t}+U_{o}h_{t-1}+b_{o}\right)$$

(5) $$c_{t}=f_{t}⊙c_{t-1}+i_{t}⊙\sigma_{c}\left(W_{c}x_{t}+U_{c}h_{t-1}+b_{c}\right)$$

(6) $$h_{t}=o_{t}⊙\sigma_{h}\left(c_{t}\right)$$

where σ denotes the sigmoid function, $f_{t}$ denotes the activation vector of the forget gate, $i_{t}$ denotes the activation vector of the input gate, $o_{t}$ denotes the activation vector of the input gate, and $h_{t}$ denotes the output vector of the LSTM unit and the initial values $c_{0}=0$ and $h_{0}=0$, the operator ⊙ denotes the Hadamard product. $W_{i},W_{f},W_{o},W_{c}$ denote the weights of the input, forget, and output gates and memory unit, respectively. Finally, $b_{i},b_{f},b_{o},b_{c}$ denote the biases of the input, forget, and output gates and memory unit, respectively [26].

At the same time, the model contains a set of frequently connected subnetworks, allowing the LSTM model to store and access information for a long time, thereby mitigating vanishing or exploding gradients. Therefore, the LSTM model has more advantages over traditional RNN algorithms, which are limited in their ability to deal with long dependencies [8].

### 2.6. The Hybrid Model—EEMD-LSTM

**Figure 2:**
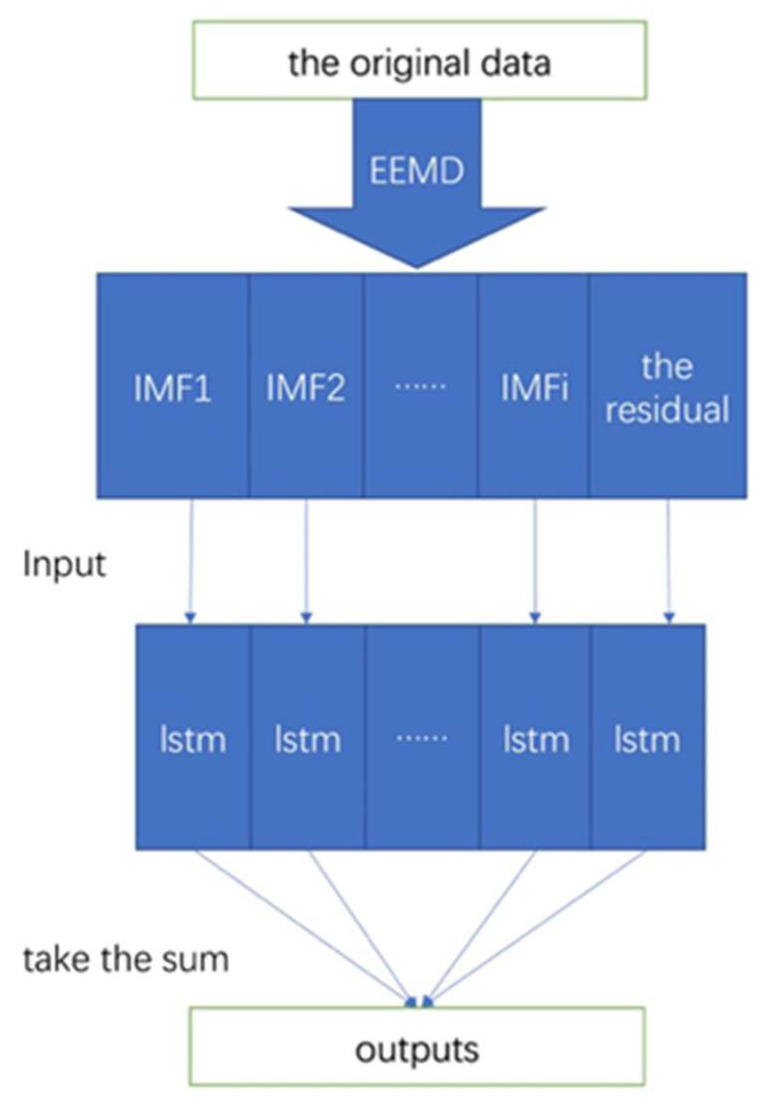
Flowchart of the proposed EEMD-LSTM hybrid model.

The EEMD and LSTM have been described previously. The LSTM may have issues with accuracy. The proposed EEMD-LSTM hybrid model leverages the strengths of EEMD and the LSTM to compensate for the shortcomings of a single model. Both EEMD and LSTM were implemented in Matlab R2020a (9.8.0.323502). The flowchart is shown in Figure 2. The proposed model was developed as follows. 

First, ensemble empirical mode decomposition was used to decompose the original epidemic data into several IMFs and a residual.

Second, the LSTM model was trained on the data, and predictions of the IMFs and residuals were individually made.

Third, the final predictions of the EEMD-LSTM model were summed to provide the final output.

### 2.7. Model Performance Evaluation

The MAPE was mainly used to evaluate the goodness of fit of the model in this study. Other indicators were used to evaluate various aspects of the model more objectively. Assuming that ${\hat{y}}_{i}$ represents the predicted value and $y_{i}$ represents the true value, we used the following four goodness-of-fit criteria to evaluate the model.

The Mean Absolute Percentage Error is given as:

$$MAPE=\frac{1}{n}\overset{n}{\underset{i=1}{\sum }}\frac{\left|{\hat{y}}_{i}-y_{i}\right|}{y_{i}}\times 100\%$$

The values range from zero to infinity: The value 0% represents a perfect model, while values higher than 100% represent an inferior model.

The Mean Absolute Error is given as:

$$MAE=\frac{1}{n}\sum_{i=1}^{n}\left|{\hat{y}}_{i}-y_{i}\right|$$

The values range from zero to infinity. Greater values denote larger errors.

The Root Mean Square Error is given as:

$$RMSE=\sqrt{\frac{1}{n}{\sum_{i=1}^{n}\left({\hat{y}}_{i}-y_{i}\right)}^{2}}$$

## 3. Results

### 3.1. The Long Short-Term Memory Model

**Figure 3:**
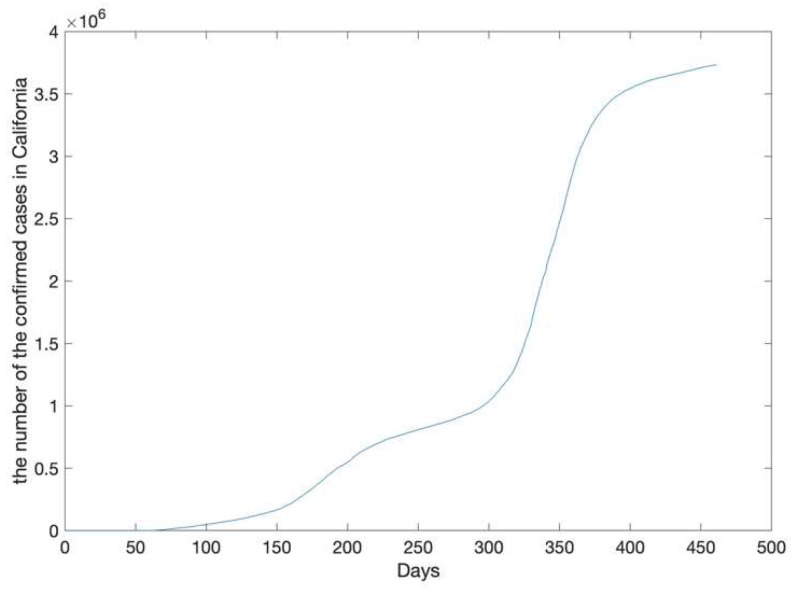
The number of accumulative confirmed cases in California.

The California epidemic data (the original sequence) from 21 January 2020, to 25 April 2021, are shown in Figure 3. The number of confirmed cases had been increasing since 21 January 2020; the increase was especially rapid after 300 days.

The California epidemic data was recorded from 21 January 2020, to 25 April 2021. The number of confirmed cases was from 0 to 3731677.

The LSTM model training data were collected between 21 January 2020, and 10 March 2021 (90%), while the data for predictions were collected between 11 March 2021, and 25 April 2021 (10%). Two LSTM hidden layers were created in this study, in addition to a dropout layer to minimize overfitting. The model used the Adam optimizer, and the maximum iteration number was set to 400. A time window was established to make better use of data for multi-step prediction, and it needed to be validated for selecting the appropriate parameter. With the test set, this study assessed the performance of models with various lag days (5, 7, 9, 11, 13, 15, 17), as shown in Table 1. The MAPE, MAE, and RMSE were minimal when the number of lag days was set to seven. It proved that the prediction model performed best for seven lag days.

**Figure 4:**
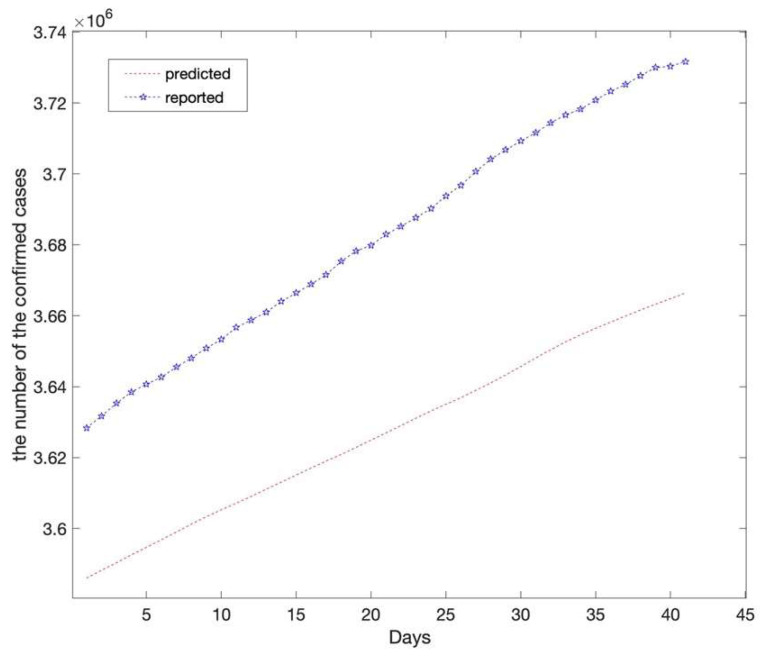
Compares the reported and predicted data (the long short-term memory model). The red dotted line depicts the long short-term memory model’s prediction results on the test set which reveals that the number of confirmed cases in California will reach 3,666,418 on 25 April 2021. The asterisk line depicts the reported number of confirmed cases in California with 3,731,677 on 25 April 2021.

Considering that the average incubation period for the novel coronavirus is approximately seven days, the lag period had to be seven days according to the actual situation and validation results. In other words, the data accrued over the first seven days affect the forecast for the next day. The predictions of the LSTM model based on the test set are shown in Figure 4. The predictions showed that the number of confirmed cases would continue to increase steadily at a relatively high rate and reach 3,666,418 on 25 April 2021. The reported number of confirmed cases in California was 3,731,677 on 25 April 2021.

### 3.2. The Proposed EEMD-LSTM Hybrid Model

**Figure 5:**
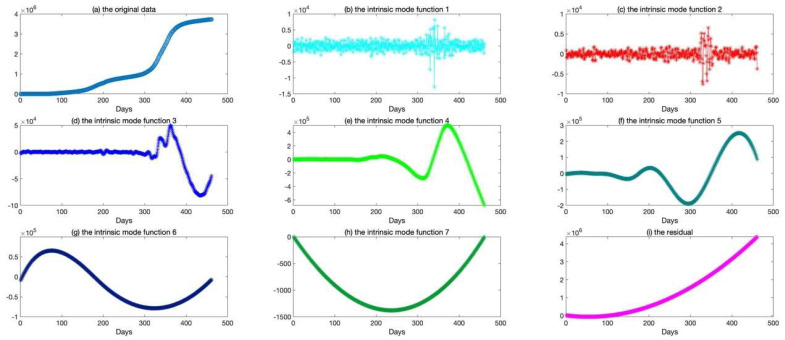
The results of the original data decomposed by ensemble empirical mode decomposition. (**a**) The picture depicts the original data (the number of confirmed cases from 21 January 2020, to 25 April 2021). (**b**–**i**) The pictures depict the decomposed sequences (IMF1, IMF2, IMF3, IMF4, IMF5, IMF6, IMF7) and the residual by ensemble empirical mode decomposition.

**Figure 6:**
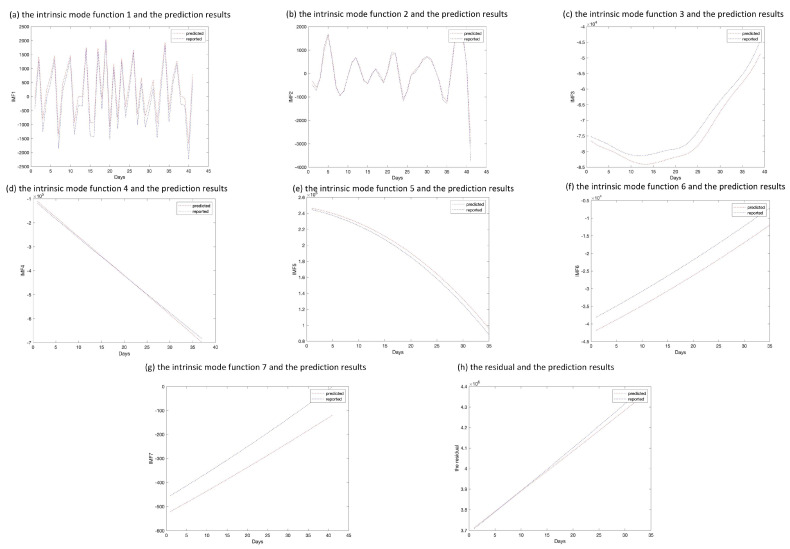
Comparison of predicted and reported values of intrinsic mode functions and residual sequence. (**a**–**h**) The pictures depict the decomposed sequences and the predicted results on the test set through the long short-term memory model.

**Figure 7:**
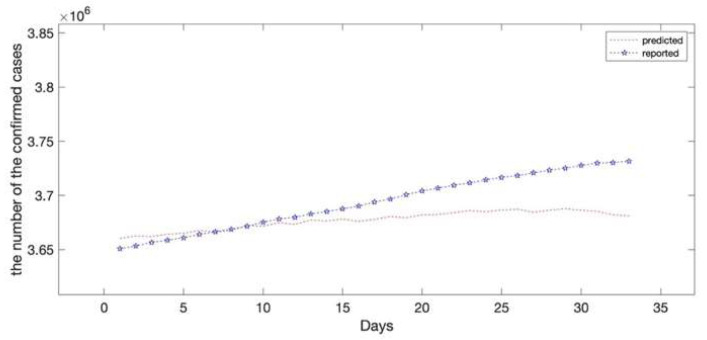
Comparisons of the reported data and the predicted data (EEMD-LSTM model). The predicted results showed that the number of confirmed cases will still increase by 25 April 2021. The red dotted line depicts the hybrid EEMD-LSTM model’s prediction results on the test set which reveals that the number of confirmed cases in California will reach 3,681,150 on 25 April 2021. The asterisk line depicts the reported number of confirmed cases in California with 3,731,677 on 25 April 2021.

The epidemic data of California were decomposed using EEMD, and the results are shown in Figure 5; the standard deviation of the decomposed white noise was set at 0.01, and the integration was performed 100 times. These decomposed sequences can be added to the original data. These sequences did not show obvious epidemiological information; however, they were easily learned by the deep learning algorithm. The decomposed sequences were used to fit the LSTM model; the results for each sequence are shown in Figure 6. The similarity of the two curves determined the reliability of the prediction. The results of the decomposed sequences were satisfactory. Finally, the predictions for each sequence were summed to obtain the final output (Figure 7). The results showed that the predicted number of cases was greater than the number of reported cases initially. Additionally, the model predicted that the number of cases would continue to increase steadily and reach 3,681,150 on 25 April 2021, which is lower than the reported 3,731,677. The results of the evaluations of the LSTM and EEMD-LSTM models are also shown in Table 2.

## 4. Discussion

COVID-19, which began to spread in 2019, continues to threaten lives, affect economies around the world, and place a burden on government hospitals. Researchers have continued to explore prediction models for COVID-19 because good predictions can provide evidence for epidemic prevention and control policies. This study combined the cutting-edge EEMD technology and an advanced deep learning model—the LSTM—to predict the confirmed cases of COVID-19 in California, the worst-hit state in the United States. Several goodness-of-fit criteria were used to evaluate the prediction model based on the test set for a more objective validation. The proposed EEMD-LSTM model combined the strengths of each model to provide more accurate predictions of the COVID-19 outbreak trends.

Ensemble Empirical Mode Decomposition is a new technology for processing non-stationary and non-linear data, and it has been successfully applied in various fields [19,20,27]. However, it is rarely used for epidemic predictions. Ensemble Empirical Mode Decomposition of data yields sequences that are more regular and suitable for model training. The data on COVID-19 confirmed cases in this work were nonlinear and non-stationary, and EEMD was applied for better predictions by subsequent models.

The SEIR and ARIMA models and other well-known statistical approaches have been widely used for predictions for the COVID-19 epidemic [4,7,11,12]. Various modifications and adaptations have been performed based on these models to boost prediction accuracy. These models were inferior to neural network models, which are more effective for learning nonlinear epidemic data. Furthermore, the LSTM model is regarded as one of the most potent deep learning frameworks for sequential data predictions and provides more benefits than conventional neural network techniques. The LSTM introduced “cells” with three gates, which addressed the limitations associated with processing long dependencies. This allows the LSTM model to store and access information over a long duration. Additionally, LSTM generalizes more effectively, which increases its usage under various conditions [8].

The predictions of the EEMD-LSTM hybrid model were more accurate than those of the standalone LSTM model, according to the results. This indicates that EEMD improved the prediction accuracy of the LSTM model. After 25 April 2021, the number of confirmed cases in California continued to increase. In this study, we present a well-fitted EEMD-LSTM hybrid model for COVID-19 prediction. One of the significant issues in data analysis is data leakage. Models may appear to be accurate due to data leakage, but they quickly turn out to not be as accurate when used for real-world judgments. When training and test data are processed illogically, such as by normalizing the entire dataset and using the test set to validate and assess the performance of the model, data leakage problems arise. In this study, normalization was applied after splitting the data sets, which seemed to undermine the prediction performance.

The number of confirmed cases kept increasing with no clear indication of a decrease on 11 March 2021. This placed a burden on the healthcare system. Furthermore, California’s policy at the time seemed to be failing to control the outbreak. The results from the hybrid model showed that the number of reported infections was higher than the model predicted, which may indicate that more aggressive measures, such as increased testing and reduced social distancing, were required at the time to contain the outbreak.

This study had limitations. The combination of EEMD and LSTM requires various strategies, such as recreating the decomposed sequences for high and low frequencies before they are inputted into the LSTM model for learning or using the decomposed sequences as multiple features to fit a multi-input LSTM model. Future research can compare the performance of these strategies for the EEMD-LSTM model based on prediction accuracy. Additionally, it can be challenging to access data such as global pandemic statistics, weather data, and social distancing. Overall, this investigation showed the usefulness of the EEMD-LSTM model for COVID-19 epidemic predictions, which may help decision-makers and guide the allocation of medical resources. The LSTM model in this study also revealed several potential future paths for more accurate prediction models.

This study has some profound implications. The government, hospitals, and other agencies can utilize this model to guide short-term projections, which will facilitate the development of realistic preventative and control measures and provision of optimal medical care for patients. It is well-known that appropriate prevention and control measures, as well as adequate medical care, can facilitate the rapid management of epidemics [28]. Reasonable preventive and control policies also alleviate the economic burden resulting from long-term lockdowns. Another model was used to predict the COVID-19 lockdowns using the normal distribution [29]. The improved LSTM model may incorporate additional parameters, such as temperature, social distance, and vaccination, which can be used to estimate changes in the environment or guide policy.

## 5. Conclusions

In conclusion, the goal of this study was to develop and validate an EEMD-LSTM hybrid model for COVID-19 prediction. A model based on LSTM, a novel technique for time series data prediction, and EEMD was used, which allowed more accurate predictions. The forecasting performance on the test set was validated using various model evaluation criteria (MAPE, MAE, and RMSE). Comparisons of the assessment criteria revealed that the proposed EEMD-LSTM hybrid model outperformed the LSTM model. The proposed model may guide epidemic supervision and control and provide a reference for a forecasting approach for future epidemics.

## Figures and Tables

**Table 1:** Evaluation of different time windows on the prediction performance.

Lag Days	5	7	9	11	13	15	17
Mean Absolute Percentage Error	0.030	0.018	0.033	0.031	0.047	0.025	0.029
Mean Absolute Error	114,048.4453	66,214.3438	123,150.7422	113,253.4453	175,371.1406	94,208.0391	109,076.2812
Root Mean Square Error	114,269.1406	66,306.9688	123,698.8281	114,207.7812	176,172.7344	94,910.125	110,076.9375

**Table 2:** Comparison of the prediction performances between EEMD and EEMD-LSTM models.

	Mean Absolute Percentage Error	Mean Absolute Error	Root Mean Square Error
LSTM	0.0151	5.58 × 10^4^	5.63 × 10^4^
EEMD-LSTM	0.0051	1.90 × 10^4^	2.43 × 10^4^

Note: Long short-term memory (LSTM), Ensemble Empirical Mode Decomposition (EEMD).

## Data Availability

The datasets analyzed during the current study are available in the Dataverse Harvard repository, https://doi.org/10.7910/DVN/HIDLTK (accessed on 14 September 2021).

## Abbreviations

ARIMA	Autoregressive Integrated Moving Average
EEMD	Ensemble Empirical Mode Decomposition
EMD	Empirical Mode Decomposition
IMFs	Intrinsic Mode Functions
LSTM	Long Short-term Memory
MAE	Mean Absolute Error
MAPE	Mean Absolute Percentage Error
RMSE	Root Mean Square Error
RNN	Recurrent Neural Network
